# Upregulation of the lncRNA *MEG3* in Metastatic Hepatoblastoma

**DOI:** 10.3390/cells15040361

**Published:** 2026-02-18

**Authors:** Morgan L. Brown, Maryam G. Shaikh, Nazia Nazam, Ali M. Eakes, Pranava Nande, Abdulraheem Kaimari, Joel C. Opara, Jamie M. Aye, Karina J. Yoon, Elizabeth A. Beierle

**Affiliations:** 1 Division of Pediatric Surgery, Department of Surgery, University of Alabama at Birmingham, Birmingham, AL 35233, USA; mlbrown6@uab.edu (M.L.B.); ameakes@uabmc.edu (A.M.E.);; 2 Division of Hematology Oncology, Department of Pediatrics, University of Alabama at Birmingham, Birmingham, AL 35233, USA; 3 Division of Cell, Developmental and Integrative Biology, University of Alabama at Birmingham, Birmingham, AL 35294, USA; kyoon@uab.edu

**Keywords:** metastasis, hepatoblastoma, lncRNA, *MEG3*

## Abstract

**Highlights:**

**What are the main findings?**

- The lncRNA *MEG3* is consistently upregulated in metastatic hepatoblastoma across cell lines, orthotopic tumors, PDX models, and patient datasets.
- *MEG3* promotes aggressive tumor phenotypes, as its silencing reduces clonogenicity, stemness, and motility, while its overexpression enhances motility.

**What are the implications of the main findings?**

- *MEG3* appears to function as an oncogenic lncRNA in hepatoblastoma, differing from its role in other malignancies.
- These findings suggest that *MEG3* may be involved in pathways associated with hepatoblastoma progression and metastasis.

**Abstract:**

Hepatoblastoma is the predominant primary liver malignancy in children, and outcomes remain poor for patients with metastatic disease. Long non-coding RNAs (lncRNAs) regulate tumor behavior, but their role in metastatic hepatoblastoma is not well defined. This study investigates the expression and functional significance of the lncRNA, maternally expressed gene 3 (*MEG3*), in a metastatic hepatoblastoma model. RNA sequencing comparing the metastatic hepatoblastoma cell line, HLM_2, with its parental HuH6 cell line identified *MEG3* as being significantly upregulated in metastatic cells. *MEG3* expression was examined using hepatoblastoma patient datasets and validated using qPCR in cell lines, orthotopic tumors, and COA67 patient-derived xenografts. The effects of siRNA *MEG3* knockdown in HLM_2 cells on clonogenicity, migration, and invasion were evaluated. The effects of *MEG3* overexpression on migration and invasion were assessed in HuH6 cells. *MEG3* was significantly upregulated in metastatic cells and orthotopic tumors compared with controls. *MEG3* silencing reduced clonogenicity, tumorsphere formation, migration, and invasion. *MEG3* overexpression increased migration and invasion. These findings indicate that *MEG3* contributes to an aggressive tumor phenotype, highlighting the need for further examination into its mechanistic role in hepatoblastoma and its potential as a biomarker or therapeutic target.

## 1. Introduction

Among pediatric patients, hepatoblastoma is the most common primary liver tumor, and its incidence continues to rise [1,2]. Up to 20% of patients have pulmonary metastases at the time of diagnosis, and the event-free survival for those with metastatic disease remains as low as 25% [3]. Treatment for these patients includes multimodal therapy with surgical resection, including liver transplantation in select cases, and cisplatin-based chemotherapy, which is associated with long-term toxicities [4]. These poor outcomes highlight the need to improve our biological understanding of metastatic hepatoblastoma.

Alpha-fetoprotein (AFP) is the most widely used clinical biomarker for diagnosis and disease monitoring. While low AFP concentration can be predictive of poor outcomes, it does not predict metastatic potential [5]. On the molecular level, hepatoblastoma is driven by genetic alterations and dysregulation of signaling pathways that promote tumorigenesis [6]. Aberrant activation of Wnt/β-catenin signaling is the most consistent molecular feature of hepatoblastoma, with activating mutations in *CTNNB1* observed in the majority of tumors [6]. Additional pathways, such as the Hippo/YAP, IGF, Notch, and TGF-β signaling pathways, have also been shown to play a role in hepatoblastoma biology [7,8,9,10,11]. However, the molecular determinants that drive metastatic progression remain incompletely characterized.

Long non-coding RNAs (lncRNAs) are RNA transcripts that are greater than 200 nucleotides. They do not encode proteins but serve a wide array of functions and regulate gene expression [12]. LncRNAs act as oncogenes or tumor suppressors depending on the context [12,13], and increasing evidence indicates that they modulate therapeutic response through epigenetic regulation, interaction with proteins, and miRNA-mediated mechanisms [14,15]. In hepatoblastoma, lncRNAs are important regulatory contributors to tumor pathogenesis; however, their roles in metastatic progression remain ill-defined [16]. Maternally expressed gene 3 (*MEG3*) is an lncRNA that has been shown to play a role in tumor metastasis [17]. In nasopharyngeal carcinoma, *MEG3* was shown to modulate migratory and invasive behavior through its regulation of sequestosome 1 [18]. Similarly, in colorectal cancer, *MEG3* has been reported to influence cell migration through miRNA-mediated mechanisms [19]. In other cancers, *MEG3* has been characterized as a tumor suppressor, where it is downregulated [17]. Some evidence has emerged demonstrating that *MEG3* is markedly upregulated in hepatoblastoma patient samples [7,20]. Given the role of *MEG3* in metastasis in other cancers and evidence of its elevated expression in hepatoblastoma, this study aimed to evaluate *MEG3* expression in hepatoblastoma metastasis employing a metastatic human hepatoblastoma cell line.

## 2. Materials and Methods

### 2.1. Cells and Cell Culture

The HuH6 human hepatoblastoma cell line was provided by Thomas Pietschmann (Hannover, Germany) [21]. HLM_2, a metastatic hepatoblastoma cell line, was previously generated using a tail vein injection model to induce pulmonary metastases from the established HuH6 cell line [22]. Both HuH6 and HLM_2 cell lines were grown under standard conditions using Dulbecco’s Modified Eagle’s Medium (DMEM, Corning Inc., Corning, NY, USA) containing 10% fetal bovine serum (FBS, HyClone, GE Healthcare Life Sciences, Logan, UT, USA), penicillin/streptomycin (1 µg/mL; Gibco, Carlsbad, CA, USA), and L-glutamine (2 mmol/L; Thermo Fisher Scientific, Waltham, MA, USA). A human hepatoblastoma patient-derived xenograft (PDX) designated COA67 was established from a male child with metastatic hepatoblastoma as previously described [22]. PDX tumor fragments were implanted subcutaneously into the flank of immunocompromised mice and underwent serial passage for maintenance, and were not passed in cell culture. Tumor tissue was dissociated using the Miltenyi Human Tumor Dissociation Kit (Miltenyi Biotec, San Diego, CA, USA). For experiments, the dissociated COA67 PDX cells were cultured in Dulbecco’s Modified Eagle’s Medium/Ham’s F12 (Corning) containing penicillin/streptomycin (1 µg/mL; Gibco), L-glutamine (2 mmol/L; Thermo Fisher Scientific), epidermal growth factor (20 ng/mL; EMD Millipore, Billerica, MA, USA), β-fibroblast growth factor (20 ng/mL; EMD Millipore), 2% B27 supplement (Gibco), and amphotericin B (2.5 µg/mL; HyClone). Cells were screened for Mycoplasma contamination using the Universal Mycoplasma Detection Kit (30-1012K, American Type Culture Collection, ATCC, Manassas, VA, USA) and were found to be free of mycoplasma infection. Cell lines were verified in the last 12 months using short tandem repeat analysis in the Genomics Core (University of Alabama at Birmingham (UAB), Birmingham, AL, USA).

### 2.2. Orthotopic Tumor Model

All animal experiments were conducted with approval from the UAB Institutional Review Board (IRB-130627006, approved 16 August 2013) and UAB Institutional Animal Care and Use Committee (IACUC-09186, approved 11 August 2025; IACUC-010133, approved 13 March 2023). Studies were performed following institutional, national, and NIH guidelines. Animals were maintained on a 12 h light/dark schedule with chow and water available ad libitum. Environmental enrichment was provided. Animals were euthanized in a humane manner in their home cages using CO_2_ and cervical dislocation.

To generate liver tumors, luciferase-positive HuH6 or HLM_2 (1 × 10^6^) cells were injected into the left lobe of the liver of 6-week-old female athymic nude mice (Charles River, Frederick, MD, USA). Animals were randomized to each experimental group using a random number generator. No experimental treatments were administered, and all animals were housed under identical conditions. No additional strategies were used to control potential confounders. Group allocation was not blinded, and investigators were aware of group allocation throughout the experiment. At four weeks following injection and weekly thereafter, d-luciferin substrate was delivered via peritoneal injection, and bioluminescence was evaluated using IVIS Lumina III and an EMCCD camera (PerkinElmer, Waltham, MA, USA) to monitor for tumor formation. Animals were euthanized after eight weeks, and tumors were harvested for study. No a priori inclusion or exclusion criteria were applied for the experimental units. All implanted animals were included in the analysis, and no animals or data points were excluded due to a lack of tumor engraftment or growth.

### 2.3. RNA Extraction, Library Preparation, and Sequencing

Total cellular RNA was extracted utilizing the miRNeasy kit (Qiagen Inc., Germantown, MD, USA) following the manufacturer’s protocol. The UAB genomics core implemented quality control, library preparation, and sequencing. RNA quality was assessed utilizing the Agilent 2100 Bioanalyzer, followed by two rounds of Poly A + selection and conversion to cDNA. The NEBNext Ultra Directional RNA Library Prep Kit for Illumina library generation kit (New England Biolabs, Ipswich, MA, USA) was used per the manufacturer’s instructions. Library quantification was performed using qPCR in a Roche LightCycler 480 with the Kapa Biosystems kit (Kapa Biosystems, Woburn, MA, USA). Sequencing was performed using the Illumina NextSeq 500 (Illumina Inc., San Diego, CA, USA) with the latest versions of the sequencing reagents and flow cells with single-end 75 bp reads.

### 2.4. RNA Sequencing Analysis

Using STAR (version 2.7.11a), the raw RNA-Seq fastq reads were aligned to the reference human genome (GRCh38 p13 Release 43) from Gencode. Transcript abundance was estimated with default parameters using Cufflinks (version 2.2.1). Using default parameters, significant changes in transcript expression, splicing, and promoter usage were determined using Cuffdiff. Significantly differentially regulated molecules were considered those with a fold change cut-off of +2 with an adjusted *p*-value of less than 0.05.

### 2.5. Patient Databases

Publicly available human hepatoblastoma transcriptomic datasets were obtained from the Gene Expression Omnibus (GEO) repository to evaluate *MEG3* expression in patient samples. The following datasets were utilized due to the availability of primary hepatoblastoma tissue and corresponding non-tumor liver controls: GSE81928, GSE51701, GSE151347, and GSE104766 [23,24,25,26]. The limma package of R software (version 4.4.3) was utilized to identify genes with |log2 fold change (FC)| > 1 and adj *p*-value < 0.01. These genes were categorized as differentially expressed genes (DEGs) between comparative groups in a pairwise manner. Volcano plots were made using Prism software (version 10.0.2) to exhibit the DEGs in all datasets.

### 2.6. Quantitative Real-Time PCR

The iScript cDNA Synthesis kit (Bio-Rad, Hercules, CA, USA) was used to synthesize cDNA in a 20 µL reaction mixture with 1 µg of RNA. SsoAdvanced SYBR Green Supermix (Bio-Rad) was used for quantitative real-time PCR (qPCR) according to the manufacturer’s protocol. The *MEG3* primer set forward 5′-CCTCTCGTCTCCTTCCTGGT-3′ reverse 5′-CACATTCGAGGTCCCTTCCC-3′ was utilized. The primer was checked for non-specific binding using the basic local alignment as previously described, which confirmed that the primer pair aligns with multiple annotated *MEG3* transcript variants, including variants 1, 2, 3, 4, 5, 6, 7, 8, 9, 10, 12, 13, 14, 15, 16, 17, 18, 19, and 20 [27]. qPCR was performed with 50 ng cDNA in a 10 µL reaction volume. PCR amplification was carried out using an Applied Biosystems 7900HT cycler (Applied Biosystems, Waltham, MA, USA) using the following cycling parameters: an initial step at 95 °C for 30 s, followed by 60-cycle amplification at 95 °C for 5 s and 60 °C for 10 s. The U6 was utilized as an internal control. U6 primer set forward 5′-GTGCTCGCTTCGGCAGCACATATAC-3′ and reverse 5′-AAAAATATGGAACGTTCACGAATTTG-3′ was used. Gene expression was calculated using the ∆∆Ct method and described as mean fold change ± SD [28].

### 2.7. Reagents

HLM_2 cells (3 × 10^5^) were transfected for 12 h prior to being utilized in experiments using *MEG3* or control small interfering RNAs (siRNAs) (ON-TARGETplus Non-targeting Pool (Catalogue #D-001810-10-20) (siNeg), Lincode SMART pool Human *MEG3* (Cat. #R-187952-00-0010) (si*MEG3* pool), siRNA *MEG3* #1 (Cat. #N-187952-01-0005), siRNA *MEG3* #2 (Cat. #N-187952-02-0005), and siRNA *MEG3* #3 (Cat. #N-187952-03-0005), Dharmacon, GE Life Sciences, Lafayette, CO, USA) at 20 nM concentration using Lipofectamine RNAiMax (Thermo Fisher Scientific) utilizing the manufacturer’s protocol. qPCR, as previously described, was utilized to confirm knockdown of *MEG3* expression prior to conducting further experiments.

### 2.8. MEG3 Overexpression Plasmid and Transfection

The *MEG3* overexpression plasmid was obtained as a generous gift from Anne Klibanski (Addgene plasmid #44727; http://n2t.net/addgene:44727 (accessed on 1 September 2025); RRID:Addgene_44727) [29]. The plasmid was sequenced for verification (Plasmidsaurus, San Francisco, CA, USA). Empty vector (pCI, #V011274) was used as a control for comparison and was prepared utilizing the manufacturer’s instructions (NovoPro Bioscience Inc., Shanghai, China). Cells were transfected using FuGENE HD Transfection Reagent (Promega, Madison, WI, USA) according to the manufacturer’s instructions. Plasmid DNA was incubated at room temperature for 15 min in OptiMEM media (Thermo Fisher Scientific) with FuGENE HD Transfection Reagent in a ratio of 3:1 of transfection reagent to DNA with 1 µg of DNA per 1 × 10^6^ cells and added to the cells. Cells were transfected for 24 h prior to use in experiments.

### 2.9. Colony Forming Assay

After performing siRNA transfection, HLM_2 cells (1 × 10^3^ cells per well) were plated in 12-well plates. After 7 days, cells were fixed and stained with 0.5% crystal violet in 20% methanol for 20 min, rinsed with deionized water, and colony numbers were assessed using ImageJ (version 1.54m) (National Institutes of Health, NIH, Bethesda, MD, USA) and the Laboratory for Optical and Computational Instrumentation (Madison, WI, USA) (https://imagej.net/ij, accessed on 2 December 2025).

### 2.10. Migration and Invasion

Migration and invasion assays were performed using a modified Boyden chamber technique. For migration assays, the bottom of Transwell inserts (8 µm pores, Corning Life Sciences, Corning, NY, USA) was coated with Type I collagen (10 µg/mL, MP Biomedicals, Santa Ana, CA, USA) for HLM_2 and HuH6 cells or fibronectin (10 µg/mL, Qiagen, Germantown, MD, USA) for COA67 cells for 4 h at 37 °C. Additionally, for invasion assays, the tops of the inserts were coated with 80 µL of Matrigel (1 mg/mL, BD Biosciences, San Jose, CA, USA) for 4 h at 37 °C. HLM_2, COA67, or HuH6 cells were placed in a 60 mm dish, and the previously described siRNA or plasmid transfection protocols were performed. After transfection, HLM_2 (1 × 10^5^), COA67 (3 × 10^5^), or HuH6 (5 × 10^4^) cells were plated on top of the insert, and 350 µL of media was placed into the well. The cells were allowed to migrate or invade for 24 h (HuH6 cells) or 72 h (HLM_2 and COA67 cells), the inserts were fixed with 3% paraformaldehyde, and stained with 1% crystal violet. Images were captured utilizing light microscopy, and cells were quantified in nine randomly selected fields per insert using ImageJ (National Institutes of Health, NIH) and the Laboratory for Optical and Computational Instrumentation (https://imagej.net/ij, accessed on 12 December 2025).

### 2.11. Tumorsphere Formation Assay

COA67 cells were treated with control siRNA (siNeg) or siRNA for *MEG3* (si*MEG3* pool, si*MEG3* #3) and plated in conditioned culture media in 96-well ultralow attachment plates using serial dilutions with 10,000, 1000, 100, 10, or 1 cell per well with 12 replicates per dilution. After 4 days, the presence of spheres in each well was determined, and extreme limiting dilution analysis (ELDA) software was used to analyze the data (http://bioinf.wehi.edu.au/software/elda/, accessed on 14 October 2025).

### 2.12. Data Analysis

All experiments were performed using a minimum of three biological replicates, and results are presented as mean ± standard deviation (SD). To evaluate statistical significance, a two-tailed Student’s *t*-test or one-way ANOVA was used as appropriate, with *p* ≤ 0.05 considered statistically significant. No formal testing of statistical assumptions was performed.

## 3. Results

### 3.1. Differential Expression of lncRNAs Between Metastatic HLM_2 and Parent HuH6 Human Hepatoblastoma Cells

**Figure 1:**
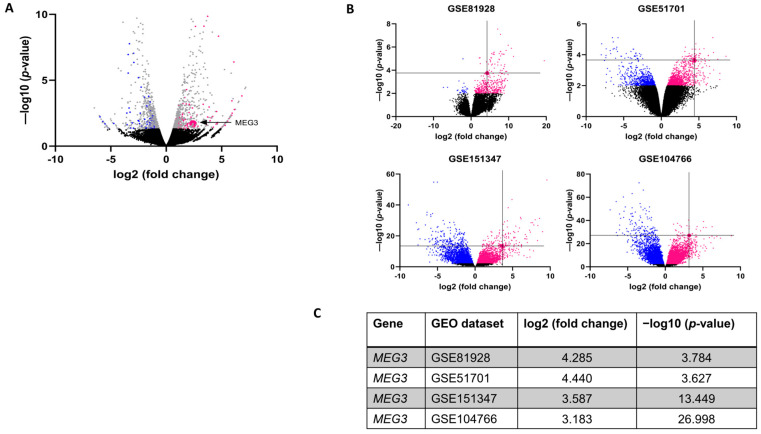
*MEG3* mRNA is increased in metastatic human hepatoblastoma cells and in human specimens. (**A**) Human hepatoblastoma cells, HuH6 (parent cell line) and HLM_2 (metastatic cell line), were examined with RNA sequencing. Volcano plot shows sequencing data with gray dots representing significantly expressed RNAs (|log2 fold change (FC)| > 1 and *p*-value < 0.05); black dots representing non-significantly expressed RNAs; magenta dots showing upregulated lncRNAs; and blue dots demonstrating downregulated lncRNAs; (**B**) Query of publicly available databases (GSE81928, GSE51701, GSE151347, and GSE104766) reveals increased abundance of *MEG3* in human hepatoblastoma tissue compared to normal liver. Intersecting black lines represent the location of *MEG3*. Magenta: upregulated RNAs; blue: downregulated RNAs; black: non-significantly expressed RNAs; (**C**) abundance of *MEG3* from datasets presented in tabular form.

Our lab previously established a metastatic hepatoblastoma cell line, HLM_2 [11]. RNA sequencing of the HLM_2 cells and the parent cell line, HuH6, was performed, and we evaluated differentially expressed lncRNAs, which were classified as those with a fold change cutoff of greater than or less than 2 with an adjusted *p*-value of less than 0.05. Sequencing identified 121 downregulated lncRNAs and 152 upregulated lncRNAs in HLM_2 cells compared to HuH6 cells (Figure 1A). We chose *MEG3* (*p* = 0.019) as the lncRNA of interest for the current investigations because it has been shown to play a role in cell migration and invasion in other cancers [30,31,32].

### 3.2. MEG3 Is Upregulated in Human Hepatoblastoma Patient Samples

Publicly available hepatoblastoma patient databases in the Gene Expression Omnibus (GEO) (GSE81928, GSE51701, GSE151347, GSE104766, Supplementary Table S1) were queried to evaluate the abundance of *MEG3* in human hepatoblastoma samples compared to normal liver [23,24,25,26]. *MEG3* was found to be significantly upregulated in hepatoblastoma tumors compared to normal liver controls in all four datasets queried. Data are presented in Figure 1B as volcano plots with down-regulated genes in blue and upregulated genes in magenta, with the cross-hairs representing *MEG3* (Figure 1B). Data are presented in tabular form in Figure 1C. Data from the full analysis are provided in Supplementary Table S1.

### 3.3. MEG3 Is Upregulated in HLM_2, Orthotopic Tumor, and Patient-Derived Xenograft (PDX) Hepatoblastoma Cells

**Figure 2:**
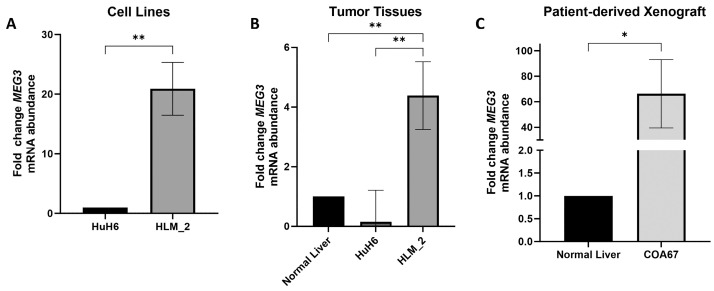
*MEG3* mRNA is increased in metastatic hepatoblastoma tumor cells, orthotopic tumors, and PDX cells. *MEG3* mRNA abundance was assessed using quantitative PCR (qPCR). (**A**) The metastatic HLM_2 cells showed significantly increased mRNA abundance of *MEG3* compared to the parent HuH6 cells; (**B**) qPCR analysis of *MEG3* expression in mouse hepatic tissue (control, no tumor), orthotopic HuH6 tumors, and orthotopic HLM_2 tumors. *MEG3* abundance was significantly increased in HLM_2 tumors compared to HuH6 tumors and non-tumor-bearing control mouse liver; (**C**) COA67 patient-derived xenograft (PDX) tumors exhibited significantly higher *MEG3* mRNA abundance compared to normal liver. Data are reported as mean ± SD and include three biological replicates. * *p* < 0.05, ** *p* < 0.01.

To confirm the sequencing findings, we performed quantitative real-time PCR (qPCR) to evaluate the mRNA abundance of *MEG3* in the HuH6 and HLM_2 hepatoblastoma cell lines. HLM_2 metastatic cells had significantly higher mRNA abundance of *MEG3* compared to HuH6 cells (Figure 2A), in concordance with the lncRNA sequencing (Figure 1A). An orthotopic tumor model was utilized, injecting HLM_2 (*n* = 3) or HuH6 cells (*n* = 3) into murine livers. Normal mouse livers were obtained for controls (*n* = 3). Orthotopic HLM_2 tumors exhibited significantly higher mRNA abundance of *MEG3* compared to orthotopic HuH6 tumors and normal liver tissue (Figure 2B). COA67 PDX tumors (*n* = 3) were harvested, and qPCR was utilized to evaluate mRNA abundance of *MEG3*. Tumors from PDX COA67, which was established from a child with metastatic disease [33], showed significantly higher mRNA abundance of *MEG3* compared to normal liver tissue (Figure 2C).

### 3.4. MEG3 Knockdown Decreases HLM_2 Cell Clonogenicity, Migration, and Invasion

**Figure 3:**
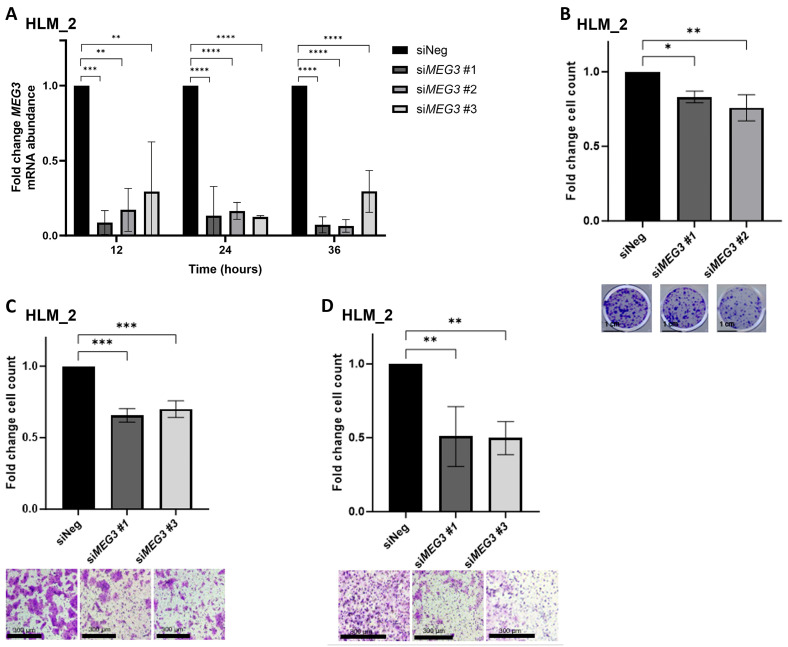
Inhibition of *MEG3* decreases clonogenicity and motility in metastatic human hepatoblastoma cells. (**A**) *MEG3* knockdown in HLM_2 cells was achieved using small interfering RNA (siRNA). qPCR was used to evaluate the expression of *MEG3* 12, 24, and 36 h after transfection with siRNA. Each *MEG3*-targeting siRNA produced a significant reduction in *MEG3* mRNA abundance at each time point compared to siNeg; (**B**) colony formation assay demonstrated decreased clonogenicity in HLM_2 cells transfected with si*MEG3* #1 and si*MEG3* #2 relative to those transfected with siNeg. Representative photos of plates are provided below the graph. Scale bars represent 1 cm; (**C**) HLM_2 cells transfected with si*MEG3* #1 and si*MEG3* #3 demonstrated significantly reduced migration compared to those transfected with siNeg. Representative photomicrographs of inserts are provided below the graph. Scale bars represent 300 µm; (**D**) invasion assay revealed significantly decreased invasion in HLM_2 cells transfected with si*MEG3* #1 and si*MEG3* #3 relative to those transfected with siNeg. Representative photomicrographs of inserts are provided below the graph. Scale bars represent 300 µm. Data are reported as mean ± SD and include three biological replicates. * *p* < 0.05, ** *p* < 0.01, *** *p* < 0.001, **** *p* < 0.0001.

Knockdown of *MEG3* in HLM_2 cells was accomplished utilizing small interfering RNA (siRNA). Three independent siRNA constructs targeting *MEG3* were utilized. A non-targeting scrambled sequence was used as a negative control (siNeg). Confirmation of target engagement was accomplished with qPCR. The mRNA abundance of *MEG3* was significantly decreased after siRNA transfection compared to the siNeg control at 12, 24, and 36 h (Figure 3A), confirming target engagement. The colony formation assay was used to evaluate clonogenicity. HLM_2 cells transfected with si*MEG3* #1 or si*MEG3* #2 were found to have significantly decreased colony formation relative to siNeg control cells (Figure 3B). Motility was assessed using a modified Boyden chamber technique. HLM_2 cells transfected with si*MEG3* #1 had decreased migration (Figure 3C) and invasion (Figure 3D) compared to siNeg control-transfected cells significantly. A second siRNA, si*MEG3* #3, was used to confirm these findings (Figure 3C,D).

### 3.5. MEG3 Knockdown Decreases COA67 Sphere Formation

**Figure 4:**
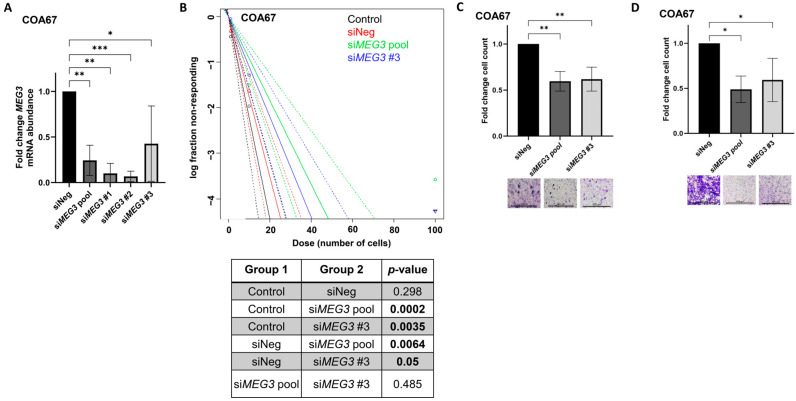
Knockdown of *MEG3* in human hepatoblastoma PDX cells leads to decreased stemness and motility: (**A**) *MEG3* knockdown in COA67 PDX cells was achieved using small interfering RNA (siRNA). qPCR was used to evaluate the expression of *MEG3* 12 h after transfection with siRNA. Each *MEG3* targeting siRNA produced a significant reduction in *MEG3* mRNA abundance compared to siNeg; (**B**) COA67 cells treated with the si*MEG3* pool and si*MEG3* #3 displayed significantly decreased tumorsphere formation compared to siNeg or non-transfected (control) cells, samples listed in the top right legend are color-coded to correspond to their respective lines in the graph; (**C**) COA67 cells treated with si*MEG3* pool and si*MEG3* #3 demonstrated significantly reduced migration compared to siNeg transfected cells; (**D**) invasion assay revealed significantly decreased invasion in COA67 cells transfected with si*MEG3* pool or si*MEG3* #3 relative to siNeg transfected cells. Data are reported as mean ± SD and include three biological replicates. Scale bars represent 300 µm. * *p* < 0.05, ** *p* < 0.01, *** *p* < 0.001.

*MEG3* knockdown was achieved in COA67 PDX tumor cells ex vivo using siRNA. Target engagement was confirmed with qPCR. siRNA-mediated knockdown significantly decreased mRNA abundance of *MEG3* at 12 h after transfection (Figure 4A). Given that COA67 cells grow in suspension, we used tumorsphere-forming capacity to evaluate clonogenicity and stemness rather than colony formation. Extreme limiting dilution analysis was utilized to evaluate the data (http://bioinf.wehi.edu.au/software/elda/, accessed on 14 October 2025). Transfection with si*MEG3* pool or si*MEG3* #3 led to decreased tumorsphere formation compared to siNeg (*p* = 0.0064, si*MEG3* pool vs. siNeg; *p* = 0.05, si*MEG3* #3 vs. siNeg) or non-transfected cells (Control) (*p* = 0.0002, si*MEG3* pool vs. Control; *p* = 0.0035, si*MEG3* #3 vs. Control) (Figure 4B), indicating a loss of stemness. siNeg transfection did not alter tumorsphere formation compared to cells without treatment (*p* = 0.298, siNeg vs. Control) (Figure 4B).

### 3.6. MEG3 Knockdown Decreases Motility in COA67 Cells

COA67 PDX tumor cells transfected with *MEG3* siRNA pool and siRNA #3 demonstrated significantly decreased migration compared to siNeg control cells (Figure 4C). Invasion assay showed significantly decreased invasion in COA67 PDX cells transfected with *MEG3* siRNA pool and siRNA #3 relative to siNeg controls (Figure 4D).

### 3.7. MEG3 Overexpression Increases HuH6 Cell Motility

**Figure 5:**
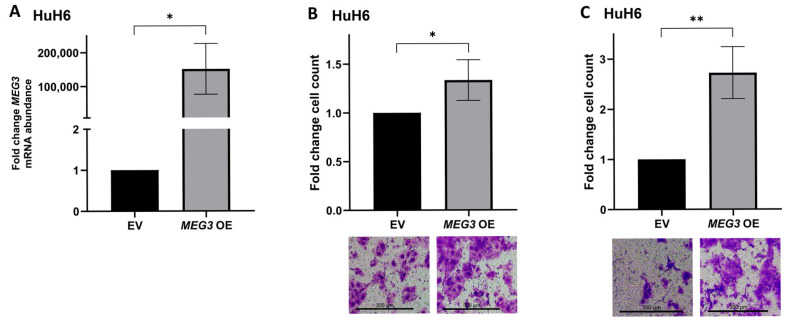
Overexpression of *MEG3* in human hepatoblastoma cells leads to increased motility. (**A**) *MEG3* overexpression in HuH6 cells was achieved using a *MEG3* overexpression (*MEG3* OE) plasmid. qPCR was utilized to determine the abundance of *MEG3* mRNA 24 h after transfection. Cells transfected with the *MEG3* OE plasmid demonstrated a significant increase in *MEG3* mRNA abundance compared to empty vector (EV) transfected cells; (**B**) HuH6 cells transfected with the *MEG3* OE plasmid demonstrated significantly increased migration compared to cells transfected with EV; (**C**) HuH6 cells exhibited significantly increased invasion when transfected with the *MEG3* OE plasmid compared to those transfected with EV. Data are reported as mean ± SD and include three biological replicates. Scale bars represent 300 µm. EV—empty vector, OE—overexpression. * *p* < 0.05, ** *p* < 0.01.

*MEG3* overexpression in the parent HuH6 hepatoblastoma cell line was achieved using a *MEG3* overexpression plasmid [29]. Target engagement was confirmed using qPCR. The mRNA abundance of *MEG3* was significantly increased in cells transfected with the *MEG3* overexpression (OE) plasmid compared to those transfected with the empty vector (EV) control (Figure 5A). Motility was evaluated with the modified Boyden chamber technique. HuH6 cells transfected with the *MEG3* overexpression plasmid demonstrated significantly increased migration (Figure 5B) and invasion (Figure 5C) compared to those transfected with the empty vector plasmid. These studies further demonstrate that *MEG3* plays a role in hepatoblastoma motility.

## 4. Discussion

Despite significant advancements in pediatric cancer treatment, survival for patients with metastatic hepatoblastoma remains as low as 25% [3]. The tumors in patients with metastasis often demonstrate more aggressive tumor biology and chemoresistance, limiting the effectiveness of multimodal treatment strategies [34]. The study of the mechanisms governing hepatoblastoma metastasis is challenging due to the limited availability of preclinical models. In fact, very few true established hepatoblastoma cell lines exist, and even fewer represent the metastatic phenotype [34,35]. Given these impediments, our lab previously developed the metastatic hepatoblastoma cell line, HLM_2, from the parent hepatoblastoma cell line, HuH6 [22], providing a novel tool to evaluate the mechanisms of hepatoblastoma metastasis.

LncRNAs have been shown to be associated with cell proliferation, migration, and invasion in pediatric solid tumors [30,31,32,36,37,38]. Specifically, in hepatoblastoma, investigators have demonstrated that lncRNAs play a central role in pathogenesis through the regulation of proliferation, apoptosis, metastasis, and therapeutic resistance [39,40]. Because of the role of lncRNAs in tumorigenicity [13,41,42] and the observation that HLM_2 cells have a more metastatic phenotype than HuH6 cells [22], we chose to evaluate the differential expression of lncRNAs between the cell lines. Of these differentially expressed lncRNAs, we filtered them according to those known to play a role in motility and tumorigenicity, thereby arriving at *MEG3* as the target of our investigations.

The lncRNA *MEG3* is located within the DLK1-*MEG3* region of chromosome 14q32.3 in humans. *MEG3* has been shown to play a role in proliferation, migration, and invasion in cancer through multiple mechanisms, including interaction with microRNAs (miRNAs), regulation of protein expression, and modulation of gene expression through binding to distal regulatory elements [17,43,44,45,46]. Heretofore, *MEG3* has primarily been considered a tumor suppressor that is downregulated in several cancer types, including gastrointestinal cancers, brain tumors, melanoma, and female organ cancers [17,45,47]. However, there are studies that have shown *MEG3* to have an oncogenic function. Sun and colleagues reported that *MEG3* is overexpressed in small-cell lung cancer (SCLC) tissues and cells and that silencing *MEG3* decreased SCLC cell viability and motility [48]. Other researchers have documented a high expression of *MEG3* in hepatoblastoma patient samples [20,49,50], hepatoblastoma cell lines, and murine hepatoblastoma liver tumors [7]. A whole-transcriptome analysis of 14 primary hepatoblastoma tumors demonstrated upregulation of genes at the 14q32 locus, including *MEG3*, compared to normal liver tissues [50]. Further, Carrillo-Reixach found that the overexpression of 14q32 locus transcripts, including *MEG3*, was associated with high-risk hepatoblastoma tumors [49]. In this study, those with a strong 14q32 gene signature and the presence of Epi-CB, a specific epigenomic cluster, were classified as high-risk tumors. High-risk tumors demonstrated a 3-year event-free survival of only 52% [49]. The association between Kagami-Ogata syndrome (UPD(14)pat) and hepatoblastoma underscores the relevance of *MEG3* dysregulation at the imprinted *DLK1-MEG3* locus [51]. *MEG3* is deleted or silenced in this disorder; however, previous studies have demonstrated *MEG3* upregulation in sporadic hepatoblastoma. These observations suggest that altered *MEG3* expression may disrupt epigenetic regulation of hepatic development and growth [51]. The findings from the current study demonstrate that *MEG3* is upregulated in metastatic hepatoblastoma. We found *MEG3* to be elevated in multiple publicly available hepatoblastoma patient datasets, and RNA sequencing analysis identified elevated *MEG3* expression in the metastatic HLM_2 cell line compared to the non-metastatic HuH6 cell line. *MEG3* upregulation was confirmed through qPCR of the metastatic and parent hepatoblastoma cell lines, as well as in PDX and orthotopic tumor models, all of which demonstrated significantly increased *MEG3* expression compared with non-metastatic controls. These findings are in line with the literature noting upregulation of MEG3 in human hepatoblastoma tumors, human hepatoblastoma cell lines, and murine hepatoblastoma tumors [7,20,49,50]. Therefore, our finding of the high expression of *MEG3* in metastatic hepatoblastoma supports a divergence from its assumed role as a tumor suppressor and underscores the need to clarify its contribution to hepatoblastoma pathogenesis.

A limitation of this study is the absence of in vivo functional validation of the role of *MEG3* in metastatic hepatoblastoma. While our findings provide mechanistic information based on in vitro and correlative analyses, direct assessment of *MEG3* function in vivo will be critical to fully establish its role in metastatic progression. Future investigations will focus on evaluating the impact of *MEG3* manipulation on metastatic behavior using stable transfected cell models in vivo. Additionally, future investigation will be critical to establish the specific mechanism of action through which *MEG3* affects the phenotype in metastatic hepatoblastoma.

Although *MEG3* has been identified in hepatoblastoma, until now, the functional implications of this lncRNA have not been explored in this tumor type. We found that *MEG3* knockdown in the metastatic HLM_2 cells resulted in reduced colony-forming capacity, indicating impaired proliferative potential. Suppression of *MEG3* also significantly decreased cellular motility, a key factor in migratory behavior associated with metastatic progression. Notably, similar phenotypic findings were observed in hepatoblastoma PDX cells established from a child with metastatic disease, where *MEG3* knockdown reduced the ability of cells to form tumorspheres and decreased motility. Conversely, *MEG3* overexpression in the non-metastatic parent HuH6 cells enhanced migratory and invasive capability, further supporting a functional role for *MEG3* in promoting aggressive hepatoblastoma cell behavior. These findings provide functional evidence that *MEG3* plays a role in the tumorigenic phenotype of hepatoblastoma and may contribute to the aggressive characteristics of metastatic disease.

## 5. Conclusions

This study demonstrates that the lncRNA, *MEG3*, is upregulated in metastatic hepatoblastoma and enhances tumorigenicity with regard to proliferation and motility in cell lines and PDX models. *MEG3* may play a role in the aggressive behavior of metastatic hepatoblastoma, and future work is needed to define the mechanisms by which *MEG3* influences tumorigenicity.

## Data Availability

The datasets analyzed and/or generated during the current study are available in the NCBI Gene Expression Omnibus (GEO) GSE81928, GSE51701, GSE151347, GSE104766, and GSE316609 (https://www.ncbi.nlm.nih.gov/geo/), accessed on 24 August 2024.

## Supplementary Materials

The following supporting information can be downloaded at: https://www.mdpi.com/article/10.3390/cells15040361/s1, Table S1.

## Abbreviations

The following abbreviations are used in this manuscript:

ANOVA	Analysis of variance
ATCC	American Type Culture Collection
bp	Base pairs
cDNA	Complementary DNA
CO_2_	Carbon dioxide
Ct	Cycle threshold
DEGs	Differentially expressed genes
DLK1	Delta-like non-canonical Notch ligand 1
DMEM	Dulbecco’s modified Eagle’s medium
EGF	Epidermal growth factor
ELDA	Extreme limiting dilution analysis
EMCCD	Electron-multiplying charge-coupled device
EV	Empty vector
FBS	Fetal bovine serum
FC	Fold change
GEO	Gene Expression Omnibus
IACUC	Institutional Animal Care and Use Committee
IVIS	In vivo imaging system
lncRNA(s)	Long non-coding RNA(s)
MEG3	Maternally expressed gene 3
miRNA(s)	MicroRNA(s)
mRNA	Messenger RNA
NIH	National Institutes of Health
OE	Overexpression
PDX	Patient-derived xenograft
Poly A	Polyadenylated
qPCR	Quantitative real-time polymerase chain reaction
RNA-Seq	RNA sequencing
SCLC	Small-cell lung cancer
SD	Standard deviation
siRNA	Small interfering RNA
STAR	Spliced Transcripts Alignment to a Reference
STR	Short tandem repeat
UAB	University of Alabama at Birmingham
